# The feasibility of universal HPV vaccination program in Shenzhen of China: a health policy analysis

**DOI:** 10.1186/s12889-019-7120-7

**Published:** 2019-06-20

**Authors:** Ruirui Chen, Eliza Wong

**Affiliations:** 1 Baoan Maternal and Child Health Hospital, Jinan University, No. 56 Yulv Road, Shenzhen, China; 2 School of Public Health and Primary Care, the Chinese University of Hong Kong, Shatin, Hong Kong

**Keywords:** HPV vaccination, Universal coverage, Stakeholder analysis, China

## Abstract

**Background:**

HPV vaccination for the prioritized adolescent girls is well accepted and implemented in developed countries as an effective measure for cervical cancer prevention and control with increasing population-level effectiveness evidence accumulated. This study is to assess the feasibility of universal HPV vaccination among adolescent girls to inform strategies to manage political dimensions of policy-making process in Shenzhen, China, offering insights for other low- and middle-income countries undergoing HPV vaccine introduction.

**Methods:**

Document review and in-depth interviews with identified stakeholders were conducted. The framework of Health Policy Triangle was adapted to guide data collection and analysis in terms of context, actors, process and content. Stakeholder analysis examining actors’ position, power, role and interest and thematic analysis focusing on data coding and theme development were used.

**Results:**

Shenzhen’s contextual factors include legislative authority under a unitary political system, economic developments and cultural values on immunization and sexuality. Stakeholders’ position and power could be explained by their role and interest in the Top-down health administration. Mothers could be potential bystanders if having little knowledge on HPV vaccination. Themes in policy-making process were problem definition, advocacy activities to promote HPV vaccination, HPV vaccine demand and access, the role of media and political attention on evidence-informed policy-making in Shenzhen. These stakeholders also discussed different aspects of program planning prospectively.

**Conclusions:**

Shenzhen witnesses a possibility of demonstration projects for local government’s horizontal accountability but no potential advocates were identified at local level for fragmented organization of public health facilities and health professionals’ lacking mobilization skills. A cervical cancer prevention expert could be a policy entrepreneur. Despite of these challenges, the recommendations to enhance the feasibility include multi-participation to engage non-governmental organizations, pharmaceuticals, target girls and their mothers, power enforcement along governing system, as well as better use of the media.

## Background

Cervical cancer is the fourth most common cancer in women worldwide with an estimated 528,000 new diagnosis and 266,000 deaths in 2012, accounting for 7.5% of all female cancer deaths and 87% of cervical cancer deaths occur in less-developed regions [1]. Since infection with oncogenic Human Papillomavirus (HPV) is a prerequisite for development of cervical cancer, controlling high frequency of infection with high-risk HPV types illustrates potential significance and benefits of vaccination efforts [2]. HPV vaccination for the prioritized adolescent girls before sexual debut is well accepted and implemented in developed countries as an effective measure for cervical cancer prevention and control. Countries including Australia, Canada, New Zealand, and the UK have succeeded in government-organized HPV vaccination programs with high uptake rate [3]. Further, early population-level impact was seen in reductions of HPV vaccine type prevalence, genital warts diagnosed and pre-cancer high-grade cervical lesions, as well as herd immunity [4–8]. World Health Organization (WHO) recommended involving HPV vaccines into national immunization program for the prioritized 9–13 year old girls in its position paper in 2014 when prevention of cervical cancer and/or other HPV-related diseases is prioritized and it is programmatically feasible with sustainable financing secured and cost-effectiveness of strategies evaluated [9].

China Food and Health Administration (FDA) approved the bivalent vaccine in July 2016, a decade after the first HPV vaccine’s licensing in the USA. The approval lag resulted from no priority-setting system in a lengthy trial registration and almost 8-year-long clinical trials done in domestic population and completed with proving evidence [10]. Due to unavailability of HPV vaccines, previous research had a focus on acceptability of different groups and economic modeling [11–14]. Given the available technical evidence, political feasibility of universal program systematically analyzed by a well-structured health policy analysis is identified as the research gap to inform policy-making on HPV vaccination, within which characteristics of HPV vaccine and detailed decision-making process can be examined [15].

Shenzhen is the Special Economic Zone of China where high level of economic development has created plenty of employment opportunities for young immigrants and migrant workers in the past two decades. Cervical cancer is seen a big public health issue because of increasing disease burden, which includes growing reported cases, estimated high-risk HPV prevalence (14%) in local sexually active women and age of women suffering cervical cancer tends to be lower [16–18]. In 2005, China launched the Program of Early Detection, Diagnosis and Treatment for Cervical Cancer and Shenzhen was chosen as one of the two demonstration sites to explore potential models for cervical cancer screening in different settings of the country [19]. Although a lot of progress has been made in cervical cancer screening, there has been no organized screening program that recruits eligible women proactively and enables them having regular screening [20]. Due to geographical closeness to and growing economic interactions with Hong Kong (HK), women in Shenzhen have unique easy access to HPV vaccination services offered by HK private clinics before legal approval of HPV vaccines in China. Therefore, Shenzhen in South China was selected as a case study and the objective of this work is to assess the feasibility of universal HPV vaccination among prioritized adolescent girls with a health policy analysis to inform strategies and tactics to manage political dimensions of policy-making process in Shenzhen, offering insights relevant for other low- and middle-income countries undergoing HPV vaccine introduction.

## Methods

### Study design and data collection

A trianglation of document review and qualitative in-depth interviews with a number of actors was used in data collection. A review of existing related documents, press releases and technical reports was conducted to offer a historical overview of events squence and valuable information about political phenomena and the context [21]. Moreover, stakeholders engaged and issues from their interactions was identified in the document review soafter an interview guide (Supplementary file) and a list of potential interviewees could be developed for this study and the coding of interview data could be verified. In-depth semi-structured interviews were carried out in line with the literature-informed guide in the period from April to July of 2016 [22–24]. Key actors who could influence the direction and outcome of the policy change to generate evidence for decision-making and operational planning were first identified to explore diverse opinions and representations according to an ecological conceptual framework in examining five interrelated but not mutually exclusive levels of influences within a complex environment: individual, interpersonal, community, institutional and public policy level [25]. Given young adolescent girls in the age group of 9–13 years may not be well informed about HPV vaccination during school education and their mothers are those who make decisions on their immunization, our study focused on four levels except individual level [26]. Purposive sampling was used to systematically select a list of participants based on the documentary data, followed by further Internet searches to exact detailed information about these stakeholders and/or their organizations. The list consisted of mothers whose daughter aged 9–13 years old, teachers at primary and middle schools, non-governmental organizations (NGOs), academics in scientific institutions, primary-level doctors, gynecologists or obstetricians, pharmaceutical companies, immunization and cervical cancer screening program coordinators in government agencies. All interviews were audio recorded and transcribed verbatim. Informed consent was received from the interviewees and ethical approval was obtained from the authors’ institute.

### Data analysis

In this prospective policy analysis, the framework of Health Policy Triangle was used for content organization and guiding data analysis in terms of context, actors, process and content in the period of problem definition, agenda-setting and policy formulation, rather than the whole process of policy-making [27]. Two specific analytic approaches were conducted within this analytical paradigm. Stakeholders’ position, power, role and interests were assessed based on their perceptions and opinions in stakeholder analysis [28]. The current level of power/influence for each stakeholder and their knowlege level was attributed on a five-point scale (1 = very low; 2 = low; 3 = moderate; 4 = high; 5 = very high) while their position was also attributed on a five-point scale (5 = high support; 4 = support; 3 = neural; 2 = opposition; 1 = high opposition). Further use of two-dimensional consideration on stakeholder’s power and position in Force-field Mapping classified stakeholders into five categories: drivers, blockers, supporters, bystanders and abstainers after identifying the main concerns of each stakeholder [29]. Thematic analysis was used to identify themes in transcripts and documentary data and the emergence of new themes was allowed to supplement pre-defined concepts and categories in the interview guide [30]. Recommendations on strategies and plans to deal with the stakeholders were further generated and discussed to assure alliances and support, design implementation strategies, and give emphasis on certain groups and future political support with appreciation of the relative importance of different stakeholders.

## Results

A total of 15 face-to-face interviews were conducted in Chinese and the length of the interviews ranged from 36 to 65 min. A summary of key findings on Shenzhen’s context and themes in policy content is presented in Table 1. Contextual factors were categorized into situational, structural, cultural and international factors to facilitate understanding the big picture of the environment [31].

**Table 1:** A summary of key findings on Shenzhen’ context and policy content

Context/Categories	Key findings
Situational factor	Recent HPV vaccine approval and availability with high demand caused by a decade’s lag
Structural factors	China’s unitary system--Through a Top-down system, National Immunization Program Department in China Center for Disease Control (CDC) disseminates administrative orders and documents including guidelines, regulations and technical proposals to provincial, municipal and district CDC along with the vertical administration system after policies or recommendations are made. The Municipal People Committee of Shenzhen has legislative authority endowed by the National People Committee as this Special Economic Zone was founded in 1980s. Regarding immunization, Shenzhen has the authority on policy-making of providing other essential vaccines in line with the epidemiological profile of local population. High-speed economic developments at the frontline of national reform from planned economy to socialist market economy. Growing needs of the citizens come with these economic developments and their voice need to be heard in this city where young immigrants and migrant workers dominate.
Cultural factors	Local population’s acceptance of immunization as prevention measures, attention on children health and increasing awareness of sexuality related issues
Exogenous influence	Impact of program implementation in early adopter countries International agencies including Bill & Melinda Gates Foundation was cited to play important roles in promoting HPV DNA testing kit and vaccine evaluation despite China is not eligible for the support from the Global Alliance for Vaccines and Immunization.
Content/Themes	Key findings
Role of schools	No precedent to immunize adolescents so effectiveness may not be achieved without school coordination but any promotion would be suspected to have a link with business benefits
Health education to adolescents and parents	Concerns on whether integrating HPV into sexual health education by health departments so educational events organized by CDC staff in parent gatherings were recommended to raise awareness
Private sector engagement	Concerns about low quality and low standard service and the capacity of these facilities is questionable to implement such a program of nonprofit nature Most of civil society organizations in China have administrative level and are framed in a similar top-down structure and their leaders are commissioned by the government. Pharmaceutical company is responsible and accountable for coping with adverse events and reimbursing sufferers as well as has an important role in surveillance strengthening.
Catch-up program	Benefits from a population-level perspective -- the catch-up program will improve the coverage of target population Vaccination age indicated in the product instruction-- crucial factor for setting vaccination age and choosing vaccine products in combination with indicators on vaccine safety and effectiveness
HPV vaccination financing	Strong measure of government commitment so the guiding principle to allocate health care resources is to focus on the population with maximized benefits. Possible strong resistance for a fully-funded program by government for HPV vaccination is not seen as a top priority on policy agenda
Program initiation	Calling for an establishment of rational understanding and assessment of HPV vaccination as an additional measure for cervical cancer prevention based on scientific evidence Policy advocacy should be taken to influence the government leaders on national priority-setting while in Shenzhen the key step was viewed to lobby someone or a certain organization

### Actors: stakeholder influence in force-field mapping

Table 2 reported the stakeholders’ knowledge level, power level, self-reported and adjusted position level, as well as their role and interest. Stakeholders in this study had various level of understanding depending on their exposure to information of research and program implementation worldwide and access to information on HPV vaccination decreases as their power level decreases. Not only power level but also medical background matters for most of the interviewees especially those out of health care system received some knowledge from a non-official pathway. A highly educated mother was approached to facilitate the communication but low awareness of HPV vaccination to prevent cervical cancer found. This may due to an age gap between young women having high attention and those having target aged daughters, regardless of education attainment.

**Table 2:** Analysis of Shenzhen stakeholders’ knowledge, power, position, role and interest

Interviewee	Stakeholder	Knowledge	Power/Adjusted	Position	Role & Interest
1	Gynecologist, district Maternal and Child Health (MCH) Hospital	3	1/1	4	Cervical screening & cancer treatment/Treatment & health education to patients
2	Public health professional, Immunization Department of Shenzhen CDC	3	4/3	4	Technical guidance on service maintenance, site management, standard operation/No interference for Category 2 vaccine
3	Official in Disease Control Department of district health bureau	3	4/3	4	Health policy-making & implementation coordination/Disease control &prevention
4	Gynecologist, Shenzhen MCH Hospital	4	No response /3	4	Cervical screening & cancer treatment & technical guidance/Treatment & health education to patients
5	Women health professional, Shenzhen MCH Hospital	4	No response /3	4	Cervical cancer screening program coordinator & technical guidance/Cervical screening & health education
6	Women health professional, National Woman & Child Health Center	5	5/5	4	Policy consultation & Technical support in policy process for MH/Woman health improvement
7	Academic & cervical cancer expert, China Cancer Fund	5	5/5	5	Academic involved in global interaction & Provision of technical support/Cervical cancer prevention & control
8	Middle school teacher	1	1/1	4	School-based program coordinator/Education & disease prevention
9	Primary school teacher	2	1/1	4	School-based program coordinator/Education & disease prevention
10	Public health professional, Immunization Department of district CDC	3	2/2	4	Implementer, trainer & supervisor/Category 2 immunization management
11	Community Health Center family doctor	4	3/2	4	Primary care, implementer & health educator/Primary care
12	Public health professional, Adult Immunization Clinic	4	3/2 or 3	4	Vaccination provision for young women/Immunization service provision
Mother 1–3	Mothers	1--3	1/1	2--4	Decision-maker for adolescent girls/Disease prevention &health improvement

**Fig. 1:**
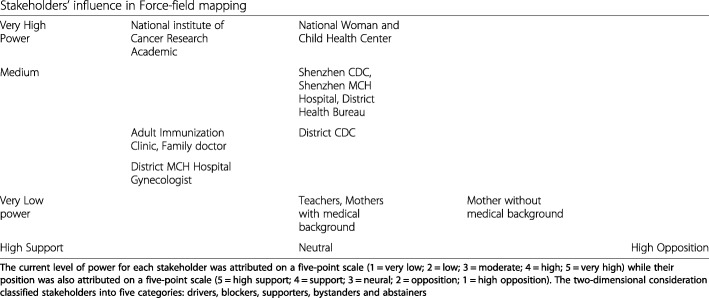
Stakeholders’ influence in Force-field mapping

Stakeholders’ position could be explained fully by their role and interest while their power level is determined by the role that they play and which level they are at in the Top-down health administration system. The public health network has been established along four administration levels: national, provincial, city and district in which disease prevention and control and maternal and child health care are two important parts. Power goes down along these levels. In the Force-field Mapping (Fig. 1) to show stakeholder’s power and position in stakeholder analysis, Interviewee 7 was classified as a driver based on the two-dimensional consideration of his high power and position level. Continuous efforts were made to raise awareness on cervical cancer prevention with his full knowledge on this issue due to his multiple-role as an academic, an international consultant and advisor of the two pharmaceutical companies. However, he preferred discussing his potential in supporting the proposed program under the role in the NGO of China Cancer Fund. He has regular communications with another national stakeholder who has technical authority in the field of women health. A technical cooperation at national level has been built to provide credible and timely information and generate a guideline for comprehensive cervical cancer prevention and control, together with the Women and Children Health Center of Peking University. They were also kept informed through interactions with international agencies like WHO and Bill & Melinda Gates Foundation.

These having intermediate level of power cannot be classified as abstainers for they supported population-level HPV vaccination if safety and effectiveness ensured. Uncertainties existed so they offered a general rather than detailed view on organization, financing, payment and service delivery of the proposal in local context. The only stakeholder (Interviewee 3) from an administrative department discussed exploring a contextualized program in the same form with pneumonia vaccination and influenza vaccination for the elderly and redefining government organization with details on local policy-making process and important procedure for success.

At municipal and district level, within-organization efforts were seen but no contact and communication between Maternal and Child Health (MCH) hospitals and Centers for Disease Control (CDC), CDCs and public hospital-run community health centers were found, subsequently no coalitions among these stakeholders as described by Interviewee 4 *“MCH Hospital and CDC are two totally different lines of disease prevention and treatment”.* The category of supporters includes the stakeholders from the adult immunization clinic, community health centers and a gynecologist for cervical cancer treatment but they all had low level of power. They have targeted different populations and these groups could overlap. Placed at low power level for policy formulation, the interviewed mothers could be representatives of a potential bystander group especially when having the responsibility to make the right decision for their daughters but little knowledge on this issue. No blockers were identified in line with this grid construction based stakeholder categorization.

### Themes in policy-making process

#### Theme 1: problem definition

Informed by disease burden indicators, cervical cancer is perceived by local gynecologists and professionals of women health and cancer prevention as a priority among women health concerns while the two stakeholders at national level concerned with social equality in under-developed areas. The other stakeholders including those without medical background have a general perception of cervical cancer as a threat to women health with increasing awareness on cervical screening for individual protection. Those in immunization system considered this form of “need-and-pay” is less effective than an organized program by government for herd immunity building.

### Theme 2: advocacy activities to promote HPV vaccination

Global initiatives of HPV vaccination were barely followed by most stakeholders except those at national level. In an international conference on HPV vaccines arranged by the WHO Asian and Pacific office in 2011, Mainland China was invited while most of the attending countries were perceived those in West Pacific where transnational drug companies provide vaccine products and financial support for program initiation. Interviewee 7, an academic on cervical cancer prevention attempted to launch the Screening Mothers and Vaccinating Their Daughter Program in less-developed areas with donated vaccines. Despite of some lobbying activities at the Ministry of Health this endeavor failed at the end for these vaccines were not allowed to enter the country before legal approval from China FDA. However, this could also be seen as the first policy advocacy on HPV vaccination at early stages of a global initiative launched by transnational drug companies.

In June 2017 when one year passed after approval but a short period before vaccine availability, a training campaign was initiated and organized by China Cancer Fund and coordinated locally by Shenzhen Maternal and Child Health Hospital. Encouragingly, the first site of this national campaign was Shenzhen and most of the interviewed professionals were involved. The aim of this campaign was to equip the medical staff in MCH hospitals, CDCs and family doctors with updated knowledge and credible information about research findings. Another focus of their efforts was to provide a platform on which different levels and disciplines of stakeholders could have direct communications.

### Theme 3: HPV vaccine demand and access in Shenzhen

Apart from procedural requirements on vaccine approval, vaccine use was also influenced by a one-year lag between approval and vaccine availability, and limited supply of imported products in early period [32]. To meet the demand, a growing number of domestic pharmaceutical companies were involved while independent research and development of new vaccines was encouraged and supported by national research funding [33]. National legislative agencies paid attention on HPV vaccine access. In a conference held by the Chinese People’s Political Consultative Conference in Peking, a representative from Guangxi Province proposed preparations for HPV vaccination during wide policy consultation. China FDA posed a reply to the proposal on accelerating HPV vaccine introduction from National People’s Congress representatives at 12th Sep of 2016 and forwarded the news about the Prime Minister’s attention on access to the latest HPV vaccine at 11th April 2018 [34, 35]. Locally, the first adult immunization clinic played a key role for young women to access vaccination services [36]. Moreover, relatively easy access to free market in HK leads to an industry of vaccination intermediary to link private clinics with interested women in Shenzhen, which also influenced the supply of vaccine products in HK and caused illegal vaccine transportation [37].

### Theme 4: role of media

Trans-border service-seeking behaviors of young women in Shenzhen were widely reported prior to vaccine approval, among which fake news on vaccine availability provided misled information. The official approval for use and market availability triggered further two rounds of intensive reporting. The first round in mid-July 2016 also included the biggest newspaper group People’s Daily, the official newspaper of the Chinese Community Party [38]. Not long after the bivalent vaccine approval, there were reports diffused on mass media about its exit from the US market, which illustrated continuous attention on the progress of HPV vaccination. The last round of reporting occurred around July 2017 when the first dose was provided in one of the early introduced provinces, which initiated new promotions through media. The importance of media guidance and management was highlighted to facilitate policy advocacy and implementation to reach the audience effectively and felt that more efforts could be put into maintaining a strategic relationship with the media.

### Theme 5: political attention on evidence-informed policy-making

Decision making process including the program framework and organization from a political perspective was perceived by some stakeholders more important than technical aspects. Other than the characteristics of HPV vaccine, attention was also paid on how to define equity in terms of political value, which serves as a basis for policy objective setting. Government commitment was considered as the most important factor in this case despite of few reactions from government officers in early policy process, reflected by no interview consent from the two relevant departments in municipal health authority. Furthermore, other concerns focus on how policy analysis on political factors could inform health policy-making in China especially in a transition period when a conflict of views exists, under the circumstance that policy-making in health field may still be a taboo subject, allowing no further discussions.

The implication of research in Shenzhen including this study was another subject of debate. Evidence from Shenzhen was perceived of little use for national policy-making and scaling-up because of different priorities. Two economic evaluations published by foreign research teams were also questioned because data from rural areas of Shanxi Province were used and generalization to other contexts should be done with caution, so these results were not suitable to inform policy-makers in Shenzhen. However, the importance of translating credible and high-quality research to policy change was identified and the implications of policy research in Shenzhen were discussed regarding the city was seen as a place where opportunities co-exist with challenges.

## Discussion

HPV vaccination policy is more than a simple technical decision made by scientific committee and health departments, which is consistent with other studies on new vaccine adoption [39, 40]. Most of advocacy activities to promote a population-level program were more likely to take place at national level. The interaction of global and domestic forces exerted the influence in the lobbying process in Peking, the political center where most important decisions are made. International agencies including WHO regional office exerted their influence through country consultations, interacting with representatives and customizing communications strategy. A sense of urgency by demonstrating health and societal cost of delays in decisions in the form of published paper was also built on a global platform [41]. A link between global and domestic efforts is a cervical cancer prevention expert and his team from the National Institute of Cancer Research. This identified policy entrepreneur, his scientific background and role as senior consultant for the two pharmaceutical companies and his networks with some relevant technocrats in government organizations offered him a privileged position at central level for future policy advocacy on HPV vaccination and priority setting at local level on this issue.

The dominant role of central government as a whole saw its coercive influence and accountability on HPV vaccine approval, importation, logistics and early supply, pricing regulation and supervision as well as self-sufficiency in vaccine and vaccine industry’s independence. Local government also has a place entitled by the horizontal accountability system in this country’s matrix institutional structure. Decentralization in policy-making has witnessed an opportunity of a demonstration project to address this issue according to the guiding principle of incrementalism in health policy-making, although there have been arguments on its function in national scale-up [42, 43]. However, no potential policy advocates were identified within health care system at local level so far. Possible explanations include fragmented organization of public health facilities that makes consensus building and controlling vested interests very difficult [44, 45], as well as local health professionals’ lack of skills and capacity in policy mobilization of relevant groups and individuals for they are not trained to be politically astute.

A variety of actors pose further challenges in their involvements. The most relevant agencies including Women’s Federation and Women and Children Working Committee have not been involved and their awareness has not been raised yet at this stage. Despite being operated within government, these departments could act as watchdogs for the interests of the target group in civil society [46]. In addition, the pharmaceutical companies should focus on their social responsibility in policy implementation rather than influencing policy formulation through rent-seeking behaviors so public transparency is needed to expose any relationships between these vaccine pharmaceuticals and other stakeholders. Wide consultation may be needed to make the voice of target girls and their mothers heard considering little evidence was found on their opinions from local studies. The influence of legislative institutions increased, indicating an opportunity to move this issue to legislative procedure. However, most municipal delegates have no substantive expertise to develop a proposal on HPV vaccination and no particular proposals were identified related with cervical cancer victims and their families. Opportunities lie in the use of the media for its important roles in wide communication and supervision of government by the public to make the government more responsive and accountable for its use of power through increasing extensive attention [45]. The recent round of extensive reporting focused on an interview with the identified policy entrepreneur and under-serving cases’ story to make appeals. A better use of this powerful tool is the key to enhance the influence for important changes are taking place in the institutional arrangements and norms of policy-making in recent China.

Corresponding to the role of the stakeholders, the enhancement of their power should go along with the governing system. Central ministries take responsibility for sectoral program development and dissemination of relevant administrative regulations and directives. China CDC is the top-level institution in public health field, so their statements in policy documents and related technical guide are important to justify this new vaccine introduction amidst competing priorities. This serves as the mainstream technical directive in the whole local political process involving political discussion and action inside health bureau and between health bureau and other stakeholders. At local level, although HPV vaccines are categorized as cervical cancer vaccine, serving as a clear policy objective for prevention, more attention is required on policy rhetoric in reframing the issue to mobilizing powerful actors within government.

Strategies to shift positions bring about the need on a general assessment of cervical cancer disease burden and the impact of an organized HPV vaccination program in Shenzhen to justify priorities. In an environment without a series of high-quality studies and local data based economic evaluations, a rapid assessment with the surveillance data in the form of brainstorming may be a practical option, within which facts and values could be argued and potential political concerns on vested and self-serving interests considered [47]. However, there is a challenge about the awareness of relevant government departments on the importance of the issue to initiate such efforts, indicating a dilemma for those concerned.

In terms of evidence-informed policy content, cultural value on sex and age of sexual debut needs to be considered when setting vaccination age. Economic evaluations were considered a technical must and well-accepted approach to inform policy-making. Furthermore, the importance of evidence use is highlighted with considerations of information source and credibility, its weight in making a decision and how to enhance the capacity for its better use because the public was exposed in massive information from different sources in this sensitive duration and perceived HPV vaccine as a medical product of limited supply and lagged progress from other parts of the world. Therefore, a neutral and professional tone should be cast by technical facilities of the government to help the public make judgments based on the available information and a united media platform should be managed by a coordinated team to have one voice on a scientific basis and avoid public distrust caused by conflicting information, especially at the stage of program initiation.

### Limitations

There are limitations inherent in the nature of the research approaches and the process to do the study. First of all, the difficulty in easily identifying and getting access to the most important stakeholders cannot enable universal views of all stakeholders. Although a large sample size is not as important as varying categories of stakeholders that should be prioritized, several important stakeholders of health ministries did not consent to the interviews so views of such policy actors could only be captured through relevant policy documents and media resources as well as engagement with other informants. Secondly, opinions of an individual stakeholder may be different from the general opinion of the group to which he belongs. As a part of a larger dynamic process in historical context, any moment of policy choice is framed by prior and later events and process. The dynamic nature of policy change indicates the fluidity of the broader policy environment so position of some stakeholders may change when more information is obtained. This study only focused on specific moments and individual events of political action and decision-making, there are risks of neglecting concomitant developments and over-emphasizing the role of actors in moving policy forward while ignoring other features and factors over time. The iterative approach of repeated stakeholder analysis is needed to supplement this single analysis representing experience at one time, which is particularly important in light of rapidly shifting political and social landscapes.

## Conclusions

This proposed HPV vaccination program is no longer a low-politics issue for the key role of government in achieving universal coverage in target population and many elements in health care system influenced to adapt this change. Despite no policy advocates were identified at local level in early stage of policy process in Shenzhen, a general assessment of disease burden and organized program feasibility is needed to justify priorities and a unified media platform is also important to disseminate information to raise awareness and ensure public transparency. The political and health care system, stakeholder constitution and engagement in policy-making as well as their power level and underlying values provide plausible explanations for a low feasibility of universal HPV vaccination in Shenzhen but opportunities existed in the engagement of NGOs, pharmaceuticals, target girls and their mothers, moving the issue to legislative procedure and better use of media in future evidence-informed policy-making process. This study has imprtant implications for those countries undergoing HPV vaccine introduction because involving multiple categories of stakeholders and assessing their position, interest and power prospectively may prove particularly useful for policy formulation with tailored strategies and enhance the likelihood of securing this policy change in their complex local context.

## Data Availability

Interview transcripts are available from the corresponding author on reasonable request.

## Abbreviations

CDC: Center for Disease Control
FDA: Food and Drug Administration
HK: Hong Kong
HPV: Human Papillomavirus
MCH: Maternal and Child Health
NGO: non-governmental organization
WHO: World Health Organization
